# Dose distribution in the thyroid gland following radiation therapy of breast cancer-a retrospective study

**DOI:** 10.1186/1748-717X-6-68

**Published:** 2011-06-09

**Authors:** S Johansen, KV Reinertsen, K Knutstad, DR Olsen, SD Fosså

**Affiliations:** 1 Institute for Cancer Research, Oslo University Hospital-Radiumhospitalet, N-0310 Oslo, Norway; 2 Department of Clincal Cancer Research, Oslo University Hospital-Radiumhospitalet University Hospital, Norway; 3 The Cancer Center, Ullevål University Hospital, N-0407 Oslo, Norway; 4 Department of Radiology, Oslo University Hospital-Radiumhospitalet, Norway; 5 Department of Physics, University of Bergen, Norway; 6 Faculty of Medicine, University of Oslo, Oslo, Norway

**Keywords:** Breast cancer, Radiotherapy, hypothyroidism

## Abstract

**Purpose:**

To relate the development of post-treatment hypothyroidism with the dose distribution within the thyroid gland in breast cancer (BC) patients treated with loco-regional radiotherapy (RT).

**Methods and materials:**

In two groups of BC patients postoperatively irradiated by computer tomography (CT)-based RT, the individual dose distributions in the thyroid gland were compared with each other; Cases developed post-treatment hypothyroidism after multimodal treatment including 4-field RT technique. Matched patients in Controls remained free for hypothyroidism. Based on each patient's dose volume histogram (DVH) the volume percentages of the thyroid absorbing respectively 20, 30, 40 and 50 Gy were then estimated (V20, V30, V40 and V50) together with the individual mean thyroid dose over the whole gland (MeanTotGy). The mean and median thyroid dose for the included patients was about 30 Gy, subsequently the total volume of the thyroid gland (VolTotGy) and the absolute volumes (cm^3^) receiving respectively < 30 Gy and ≥ 30 Gy were calculated (Vol < 30 and Vol ≥ 30) and analyzed.

**Results:**

No statistically significant inter-group differences were found between V20, V30, V40 and V50Gy or the median of MeanTotGy. The median VolTotGy in Controls was 2.3 times above VolTotGy in Cases (ρ = 0.003), with large inter-individual variations in both groups. The volume of the thyroid gland receiving < 30 Gy in Controls was almost 2.5 times greater than the comparable figure in Cases.

**Conclusions:**

We concluded that in patients with small thyroid glands after loco-radiotherapy of BC, the risk of post-treatment hypothyroidism depends on the volume of the thyroid gland.

## Introduction

Hypothyroidism has been reported as the most common thyroid disease following radiotherapy (RT) to the neck in patients with Hodgkin's lymphoma and head and neck tumors. In such patients the whole or large parts of the thyroid gland are located within the target volume and are irradiated at high-dose levels [1-10]. Based on this experience the adult thyroid gland is viewed as a relatively radiation-resistant organ though the range of thyroid-ablative radiation doses seems to be wide, being 10-80 Gy according to Floo et al. [11]. The association between RT and hypothyroidism in breast cancer (BC) patients has been investigated in only a few studies [12-16]. On the other hand, radiation exposure to parts of the thyroid gland seems unavoidable in BC patients receiving RT to the ipsilateral supraclavicular fossa. Joensuu et al. [12] demonstrated that 17 of 80 patients (21%) had developed thyroid hypofunction 7 years after postoperative loco-regional RT for BC. Bruning et al. [13] concluded that hypothyroidism was significantly more frequent in BC patients who had received irradiation to the supraclavicular lymph nodes compared to non-irradiated BC patients.

Although the risk of radiation-induced hypothyroidism in BC patients probably is small, it is of interest to explore the relationship between radiation exposure and thyroid function in BC patients. Theoretically the development of hypothyroidism in these patients would primarily depend on the volume receiving relatively high radiation doses (≥ 30 Gy) thus with the risk of insufficient post-radiotherapy hormone production. This volume may show considerable inter-patient variation, as the size of the thyroid gland may vary from patient to patient. However, to our knowledge, no study has evaluated the association between the thyroid volume exposed to high-dose irradiation and the development of post-RT hypothyroidism in BC.

In the present explorative case-control study, we compared findings from thyroid dose volume histograms (DVHs) in 16 breast cancer patients with post-radiotherapy (post-RT) hypothyroidism with 16 similarly treated patients without this late-effect, all patients being followed up after a median of 4 years after their breast cancer diagnosis. The primary aim was to calculate each patient's absolute volume of the gland receiving a defined dose and to compare the findings between Cases and Controls.

## Patients and methods

In 2003/2004, 415 women treated with RT at the Norwegian Radium Hospital during the years 1998 and 2002, were invited to take part in a follow-up study assessing long-term treatment effects [14]. All had had surgery for stage II/III breast cancer (BC) consisting of modified radical mastectomy (MRM) or lumpectomy (BCS: breast conserving surgery) and axillary lymph dissection, and most patients received chemotherapy and Tamoxifen.

Women considered for the study were identified by the hospital's radiotherapy registry and fulfilled the following inclusion criteria i) Adjuvant radiotherapy to the chest wall and the regional lymph node stations, ii) age ≤ 75 years in 2004, iii) no recurrence of breast cancer, and iv) no other cancer except for basal cell carcinoma, carcinoma in situ of the uterine cervix, or prior or simultaneous surgery for contralateral breast cancer stage I treated with surgery only v) no pre-BC hypothyroidism or nodular goiter. The follow-up study consisted of a mailed questionnaire and an out-patient examination at the Norwegian Radium Hospital. Out of 318 patients who both completed the questionnaire and attended the out-patient examination, 207 had received RT based on CT dose planning (CT-RT), and patients included in the present study were all treated with the same CT-RT.

All BC patients attending the survey had blood samples drawn for evaluation of thyroid function (TSH, T3 og T4). However, for our sub-study, results from these tests were not taken into consideration as majority of the included patients reporting to have hypothyroidism also received "Thyroxin". This drug results in normalization of the thyroid function in blood test. Starting the use of this drug was interpreted as a confirmation of hypothyroidism.

Cases were thus women who, according to self-report in their questionnaires and the assumed routinely taken blood test, had no pre-BC hypothyroidism, but started their thyroxin replacement therapy. Controls were identified among woman participating in the survey, consisted of 16 breast cancer patients with no pre-BC hypothyroidism and without a history of post-treatment hypothyroidism according to their normal blood test before survey, self-reported medical history and self report. For each Case one control was found who as much as possible matched the Case concerning age, stage at presentation and treatment.

None of the 32 included patients in our study had ever undergone thyroid surgery.

### Radiotherapy

All women were treated with 4-field RT in which the target volume included the breast (after BCS) or the chest wall (after MRM), the ipsilateral supra-and infraclavicular fossa, ipsilateral lymph nodes along the internal mammary artery and ipsilateral axilla. The RT planning was based on transverse CT scans covering the region from the 6th cervical vertebra to the middle part of the abdomen. CT slice thickness and pitch was 1.0 cm. The clinical target volume, both lungs and the heart, but not the thyroid gland were routinely delineated in the planning CT images. Treatment planning and dose calculation were performed using the Helax-TMS (Version 6.0 or higher) system applying a Pencil Beam algorithm. The voxel size in the dose calculation matrix was 0.5 × 0.5 × 0.5 cm^3^.

**Figure 1:**
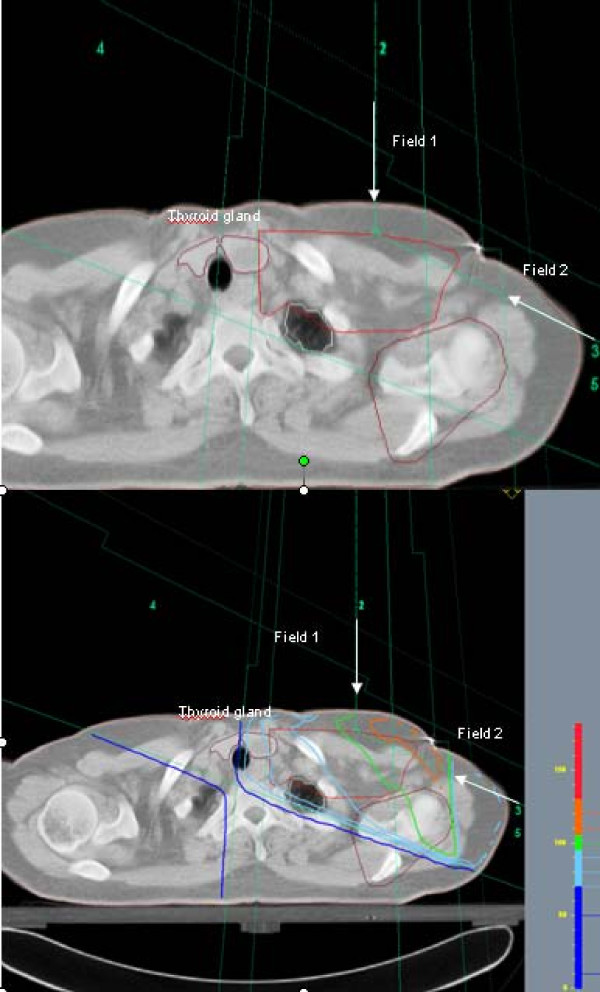
**Oblique (axillary field) (field 2) and supraclavicular (field 1) fields used in CT-RT**. The location of the thyroid gland within the axillary beam is illustrated. The thyroid gland is pink colored. The same figure with and without isodose lines is shown.

The beam arrangement consisted of 4 half-beams with two tangential beams covering the caudal part of the target volume, and one anterior-posterior field (0°) and one oblique field, typically 110-115°, covering the cranial part of the chest wall (Figure 1). The beam angles, apertures, weights and dynamic wedges were optimized by standard (forward) planning. The photon beams energy was 6 MV using a Varian Clinac (Varian Medical System) linear accelerator. The dose plans were normalized to the mean dose to the planning target volume (PTV).

The breast/chest wall should receive a total dose of 50 Gy, and the regional lymph nodes 46-50 Gy. Six of the women received an additional boost of 10 Gy to the tumor bed (9 or 12 MeV electrons using a circular field with a diameter of 5-9 cm, not included in the CT-based treatment planning).

For the purpose of the current study a radiologist delineated the thyroid gland on the planning CT-images of the Cases and Controls, and the individual volume of the gland was calculated (VolTot [cm^3^]). Based on each patient's DVH the volume percentages of the thyroid absorbing respectively 20, 30, 40 and 50 Gy were then estimated (V20, V30, V40 and V50) together with the individual mean thyroid dose over the whole gland (MeanTotGy). Subsequently the absolute volumes (cm^3^) of the thyroid gland receiving respectively < 30 Gy and ≥ 3 0 Gy were calculated (Vol < 30 and Vol ≥ 30). The 30 Gy dose was taken as the point of influencing the development of hypothyroidism, as the median of MeanTotGy was observed to be 31 Gy in both Cases and Controls in the present study.

### Statistics

To assess differences between Cases and Controls, non-parametric Mann-Whitney test were employed. The choice of the statistic tests are dependent generally on whether the data were normally distributed or not. A P-value < 0.05 was considered to be statistically significant.

### Ethics

All patients provided a written consent form to participate in the study, which was approved by the Ethical committee of the Health Region South and the Data Inspectorate of Norway.

## Results

Table 1 confirms the comparability of the 16 Cases and the 16 Controls as to age, observation time, initial stage, surgery and systemic treatment as well as the adjuvant radiotherapy. Median time from BC diagnosis to the survey was 44 months in both Cases (38-56) and Controls (37-56).

**Table 1:** Individual and Overall

Characteristic	Cases	Controls
**Age^1^**(yrs)		
< 50	3	3
> 50	13	13
Median	56	56
Range	44-75	43-73

**Obstime**^2 ^**(months)**		
< 50	11	12
≥ 50	5	4
Median	44	44
Range	38-56	37-56

**Stage**		
II	13	13
III	3	3

**Surgery^3^**		
MRM	12	12
BCS	4	4

**Chemo^4^**		
FEC	11	11
other	1	1
No	4	4

**Number of Cycles**		
6	11	11
4	1	1

**Tamoxifen**		
yes	15	15
No	1	1

**RT (Gy)**		
50 Gy	13	13
50+10 Gy	3	3

Radiotherapy, surgical and chemotherapy treatment characteristics for cases (group A) and controls (group B).

^1^**Age**: Age at follow-up

**^2^Obstime**: observation time from breast cancer diagnosis to the outpatient examination

**^3^Surgery: **MRM = Radical mastectomy, BCS = breast conserving surgery

**^4^Chemotherapy: **FEC = (5Fluoro-Uracil, epirubicin, cyclophosfamide), FEC- regimen

Other = 4 cycles of epirubicin

No = no chemotherapy

No statistically significant inter-group differences were found between V20, V30, V40 and V50Gy or the median of MeanTotGy (Table 2A and 2B, the latter being 31 Gy in both Cases and Controls), if combining both groups. In contrast, in Controls the median VolTotGy was 2.3 times above median VolTotGy in Cases (ρ= 0.003), with large inter-individual variations in both groups. As a consequence the volume of the thyroid gland receiving ≥ 30 Gy in Cases was almost 2.2 times less than the comparable figure in Controls (ρ = 0.001). Further, among Controls the thyroid volume receiving < 30 Gy was 2.5 times greater than the comparable figure among Cases (ρ = 0.000).

**Table 2:** A: Total thyroid volume (cc) and thyroid dose volume data (%) for dose levels 20, 30, 40 and 50 Gy and for mean thyroid dose.

**A: Total thyroid volume (cc) and thyroid dose volume data (%) for dose levels 20, 30, 40 and 50 Gy and for mean thyroid dose**.								
**Case no**.	**VolTot(cm3)**	**V20(%)**	**V30(%)**	**V40(%)**	**V50(%)**	**MeanTotGy**	**Vol ≥ 30(cm3)**	**Vol < 30(cm3)**

1	10	95	75	49	11	38	7	2
2	8	93	70	37	0	28	5	2
3	11	92	71	43	6	31	8	3
4	7	97	76	47	10	39	6	2
5	9	100	83	53	12	41	7	2
6	3	95	74	43	4	29	2	1
7	7	94	68	33	0	31	5	2
8	5	88	64	31	0	29	4	2
9	8	89	66	34	0	31	6	3
10	8	89	64	30	0	22	5	3
11	2	94	63	19	0	25	1	1
12	3	91	59	13	0	22	2	1
13	5	90	66	33	0	32	3	2
14	6	90	66	33	0	31	4	2
15	5	89	61	24	0	25	3	2
16	7	94	70	37	0	42	5	2

**Overall**								

All:	Median:	Median:	Median:	Median:	Median:	Median:	Median:	Median:
16	7	93	67	34	0	31	5	2
	Range:	Range:	Range:	Range:	Range:	Range:	Range:	Range:
	1.9-11.4	88-100	59-83	13-53	0-12	22-42	1.2-8.1	0.7-3.3

**B: Total thyroid volume (cc) and thyroid dose volume data (%) for dose levels 20, 30, 40 and 50 Gy and for mean thyroid dose**.								

Control no.	VolTot(cm3)	V20(%)	V30(%)	V40(%)	V50(%)	MeanTotGy	Vol ≥ 30(cm3)	Vol < 30(cm3)

1	33.4	91.0	72.0	46.0	10.0	33.0	24.0	9.4
2	16.1	95.0	77.0	51.0	16.0	34.9	12.4	3.7
3	40.1	94.0	74.0	46.0	11.0	33.4	29.7	10.4
4	12.8	92.0	70.0	38.0	0.0	32.3	9.0	3.8
5	10.6	90.0	69.0	40.0	4.0	31.6	7.3	3.3
6	6.1	89.0	62.0	26.0	0.0	28.2	3.8	2.3
7	21.8	90.0	66.0	35.0	0.0	28.9	14.4	7.4
8	2.7	92.0	73.0	46.0	9.0	31.1	1.9	0.7
9	18.4	94.0	74.0	46.0	10.0	33.0	13.6	4.8
10	19.2	89.0	66.0	35.0	0.0	29.4	12.7	6.5
11	11.9	91.0	65.0	31.0	0.0	30.3	7.7	4.2
12	16.8	86.0	64.0	33.0	0.0	28.2	10.8	6.0
13	10.6	92.0	72.0	40.0	0.0	37.6	7.6	3.0
14	16.9	90.0	70.0	43.0	8.0	31.3	11.8	5.1
15	12.8	89.0	66.0	34.0	0.0	28.8	8.5	4.3
16	41.0	93.0	70.0	39.0	0.0	32.7	28.7	12.3

**Overall**								

All:	Median:	Median:	Median:	Median:	Median:	Median:	Median:	Median:
16	16	91	70	40	0	31	11	5
	Range:	Range:	Range:	Range:	Range:	Range:	Range:	Range:
	2.66-41	86-95	62-77	26-51	0-16	28-38	1.9-29.7	0.7-12.3

A. Cases: Individual and Overall. B. Controls: Individual and Overall.

## Discussion

In this case-control study, breast cancer patients who developed post-RT hypothyroidism displayed significantly smaller thyroid glands volume before the adjuvant radiotherapy than their controls who had not developed post-RT hypothyroidism. This resulted in significantly smaller absolute thyroid sub-volumes receiving ≥ 30 Gy in Cases than in Controls. The median of the individual mean thyroid dose was 31 Gy [22Gy-42Gy] in Cases. The relatively small volumes with high radiation exposure may be responsible for the post-radiotherapy development of hypothyroidism in Cases. Compared to their Controls, Cases were after radiotherapy left with smaller thyroid volumes which were enabled to produce sufficient amount of hormone.

When estimating the incidence/prevalence of post-RT hypothyroidism, it is important to separate clinical symptomatic hypothyroidism from biochemical hypothyroidism. As screening for thyroid function has not been a routine in breast cancer survivors, we believe that our BC Cases presented to their family doctor clinical symptoms compatible with decreased thyroid function which resulted in the diagnosis of hypothyroidism. In the study of Reinertsen et al. [14] an increased prevalence of hypothyroidism (18%) in breast cancer patients was observed compared to 6% the prevalence in the general population in Norway. The difference is related to a higher incidence after breast cancer treatment.

According to the literature both age and radiation dose are related to development of post-radiation hypothyroidism. Radio sensitivity of the thyroid gland is believed to decrease with increasing age. Bonato and colleagues [15] showed that 23 of 59 childhood cancer survivors developed biochemical hypothyroidism after radiotherapy to the head and neck as well as total body irradiation. A median thyroid dose in Bonato et al.'s study was 42 Gy (inter-quartile range: [27-72Gy]) [15]. After high-dose radiotherapy the 5 years incidence of biochemical hypothyroidism was 48% in adults with head and neck cancer [17]. However, in another study carried out by Smith et al. [16] the 5 years incidence of thyroxin requiring hypothyroidism in 38.255 irradiated and non-irradiated women older than 65 years diagnosed with breast cancer and 111.944 cancer-free controls was identified. Their results showed an identical 14% incidence of hypothyroidism development in both irradiated patient group and non-irradiated [16]. Emami et al. [18] suggested a tolerance dose of 45 Gy leading to development of clinical hypothyroidism in 8% of the individuals followed for 5 years after completion of radiotherapy with 45 Gy. Yoden et al. [19] have suggested that the percentage volume of the thyroid gland receiving doses between 10-60 Gy (V10-V60) would represent a predictor of hypothyroidism. According to Yoden et al. [19] V30 Gy had a significant impact on the peak level of TSH. Other estimations of incidence after 50 Gy applied to the whole thyroid gland [10-80Gy] range from 2% - 50% [20,21]. After head and neck irradiation doses of 10-80 Gy to the thyroid are reported to lead to dysfunction of the gland [11]. The diversity of these figures illustrates that the threshold for thyroid radiation and development of hypothyroidism is not clear. The admittedly small present study emphasizes the role of the individual thyroid gland volume for the development of post-radiotherapy hypothyroidism in BC patients. The sub-volume receiving ≥ 30 Gy seems to determine whether or not sufficient thyroxin is produced after radiotherapy. Among Cases the total thyroid volume and the sub-volume receiving ≥ 30 Gy are sufficiently smaller than in Controls. Interestingly Bonato et al. [15] confirmed in their study that hypothyroid individuals had smaller glands than those with normally functioning glands, though it is not quite clear on the report whether volume measurements have been performed before radiotherapy or afterwards in connection with the reported survey.

The measurement of the thyroid gland volume represents the main limitation of this small study. On the background of the lack of contrast the delineation of the individual thyroid gland on the CT images remained a constant difficulty even for an experienced radiologist. Using ultrasonography, larger volumes have been described [22] than accomplished in our study. However, thyroid gland sizes ranging from 3.6-6 cm in length, 1.5-2 cm in width and 1-2 cm depth are reported [23], which are more in agreement with our study. Finally, as our findings are principally based on the selective differences between the gland volumes in Cases and Controls, any systematic measurement error is of less importance, provided that its similar presence in Cases and Controls.

The impact of adjuvant chemotherapy and hormone treatment on the risk of hypothyroidism among patients with head and neck malignancies is investigated by both Kanti et al. [24] and Sinrad et al. [25]. These authors found no effect of adjuvant chemotherapy on thyroid gland function, though chemotherapy for head and neck cancer differ from that applied in BC patient group. Also, Jereczk-Fossa and colleagues [20] have concluded that the impact of chemotherapy and endocrine treatment on the risk of hypothyroidism is still controversial. The significant difference in thyroid size between Cases and Controls in the current study and the high similarity of systemic treatment in all Cases and Controls of our study makes it impossible to analyse the impact of chemotherapy on post-BC hypothyroidism development.

We concluded that patients with small thyroid glands are at particular risk to develop hypothyroidism after radiotherapy for breast cancer, as less tissue with radiation doses less than 30 Gy is available for sufficient thyroxin production. Further investigations in larger cohorts are required to confirm our results.

## Competing interests

The authors declare that they have no competing interests.

## Authors' contributions

All authors read and approved the final manuscript. SJ wrote the paper and performed the dosimetric and statistical analysis. KVR prepared the patient material and participated in the discussion of the results. KK delineated the thyroid gland. DRO participated in the coordination of the study. SDF carried out the design of the study and edited the manuscript.
